# Seroprevalence and risk factors for Kaposi’s Sarcoma associated herpesvirus among men who have sex with men in Shanghai, China

**DOI:** 10.1186/s12879-023-08028-y

**Published:** 2023-01-31

**Authors:** Yi Li, Xingcan Zhang, Yue Zhang, Minqi Wei, Sijie Tao, Ying Yang

**Affiliations:** 1 Department of Epidemiology, School of Public Health, Fudan University, Shanghai, China; 2 Department of Gynecology, Obstetrics and Gynecology Hospital, Fudan University, Shanghai, 200011 China; 3 Department of HIV/AIDS Prevention, Center for Disease Control and Prevention of Minhang District, Shanghai, 201101 China

**Keywords:** KSHV, MSM, Risk factor, China

## Abstract

**Background:**

This study aimed to facilitate the understanding of the transmission route and risk factors that might contribute to the infection of Kaposi’s sarcoma associated herpesvirus (KSHV) among men who have sex with men (MSM).

**Methods:**

A cross-sectional study of 520 subjects was conducted in Shanghai, China in 2020. Plasma samples were collected and screened for KSHV, HIV, HBV, HCV, and syphilis. Univariate and multivariate logistic regression analyses were conducted to explore potential correlates of KSHV infection.

**Results:**

The overall seroprevalence of KSHV was 43.8%, with an adjusted value of 29.8% according to the sensitivity and specificity of the KSHV screening assay. Individuals with lower levels of monthly income (Chi-sqaure_*trend*_ = 4.11, *P* = 0.043) and more male sex partners (Chi-sqaure_*trend*_ = 6.06, *P* = 0.014) were more likely to be infected with KSHV. Also, KSHV seropositivity was positively associated with being a student (aOR = 1.96; 95%CI: 1.09–3.61), being coinfected with HCV (aOR = 2.61; 95%CI: 1.05–7.10), and syphilis (aOR = 2.91; 95%CI: 1.30–6.89).

**Conclusions:**

The prevalence of KSHV in MSM remains high. As a risky sexual behavior, having multiple male sex partners is a key contributor to KSHV infection among this population. Efforts designed to control modifiable risk factors in order to reduce the burden of KSHV infection are indispensable. High KSHV seroprevalence among students MSM deserves more attention.

**Supplementary Information:**

The online version contains supplementary material available at 10.1186/s12879-023-08028-y.

## Background

Kaposi’s sarcoma associated herpesvirus (KSHV), also termed human herpesvirus type 8 (HHV-8), has been considered as the etiologic agent for Kaposi’s sarcoma (KS), primary effusion lymphoma (PEL), multicentric Castleman’s disease (MCD) and KSHV-associated inflammatory cytokine syndrome (KICS) [1–4]. The seroprevalence of KSHV infection differed considerably across regions and subpopulations [5–7]. In endemic districts such as Uganda, KSHV seroprevalence was found to be as high as 50% in the general population, while in non-endemic areas such as the USA, the seroprevalence of KSHV infection was estimated at 6% or lower. Men who have sex with men (MSM), a traditional high-risk group for HIV and sexually transmitted infections (STIs), were also perceived to be alarmingly vulnerable to KSHV infection. Across non-endemic areas, the highest seroprevalences of KSHV were recorded in both HIV-positive MSM (30–60%) and HIV-negative MSM (20–30%) [8–11]. HIV infection was not only associated with KSHV infection, but also a promoter of KSHV-associated malignancies [12, 13]. It is important to note that symptomatic episodes or disease progression following KSHV infection can be devastating, especially in immunocompromised individuals. Given the high risks and potential adverse outcomes of KSHV infection among MSM, it is necessary to identify modifiable risk factors for KSHV transmission and curtail its epidemic spread in this population.

The transmission route and specific risk factors for KSHV infection among MSM remain controversial. Sexual route has been considered to play a key role in KSHV transmission among this high-risk population [14–16]. According to research, receptive anal or oral sex may increase the risk of KSHV infection [14, 17]. Saliva was reported as the highest shedding place for KSHV; in endemic areas, oral exposure to infectious saliva was considered to be most likely the major source of KSHV infection [18]. Notably, the use of saliva as a lubricant in anal sex appears to favor the spread of KSHV among MSM [19]. As an aside, demographics (age, race), risky behaviors (recreational drug use, multiple sex partners), and STIs such as syphilis were also thought to be associated with KSHV infection [14, 20–22]. However, it is noteworthy that there is no consensus on all these findings.

Shanghai, one of the most developed and prosperous metropolises in China, is more open to diversity and more inclusive compared with other cities in the country, attracting numerous MSM migrants and possessing a large MSM population [23, 24]. Understanding the burden of KSHV infection is particularly important for strategic health planning. Therefore, we conducted a study in this city to investigate the seroprevalence and potential correlates of KSHV infection among a sample of MSM and further facilitate the understanding of the KSHV transmission route in this group.

## Methods

### Study site and participants

The present study was conducted from January 2020 to October 2020 in Shanghai. We recruited MSM from gay-oriented venues such as bars, night clubs, teahouses, bathhouses, and saunas. To be eligible to participate in the study, a participant must be a male who: (1) was aged 18 or above; (2) had a lifetime experience of ever having had sex with another man; and (3) was able to provide written informed consent.

### Data collection

An anonymous questionnaire interview was conducted for all participants to collect information on sociodemographic characteristics and sexual behaviors in the past 6 months of study participants. Interviews were administered face-to-face by trained public health workers in a private room. Completed questionnaires were placed in a large bag containing other completed questionnaires to reassure the participants that no one could identify the participants’ responses. Each participant received a monetary incentive of 50 RMB after the survey.

### Sample collection

Venous blood was collected by experienced health-care workers using sterilized needles and tubes and then transferred to the laboratory within 2 h after collection while being maintained at 4 °C. Plasma specimens were stored at − 80 °C until being tested. Each specimen was coded by a unique identification number and was analyzed by two professional technicians blinded to the personal identity of the study participants.

### Laboratory methods

#### KSHV testing

An immunofluorescence assay (IFA) was performed on plasma samples as previously reported [25]. Briefly, BC-3 cells (KSHV positive and Epstein-Barr virus negative B cell line, American Type Culture Collection, Manassas, VA), stimulated by tetradecanoyl phorbol acetate (TPA), were fixed and permeabilized and used for monoclonal antibody-enhanced IFA. A sample was characterized as KSHV seropositive only if it was positive at a standard serum dilution of 1:40. Slides were independently read by two experienced laboratory workers to reduce subjectivity in status assignations.

#### HIV testing

Serum samples were screened for HIV antibody using an enzyme-linked immunosorbent assay (ELISA; Abbott Laboratories, Chicago, IL, USA) according to the manufacturer’s instructions. Positive samples were confirmed by a western blot assay (HIV BLOT 2.2; Genelabs Diagnostics, Singapore) [26].

#### HBV testing

HBsAg was tested using an ELISA kit (Wantai Biotech Pharmacy Enterprise Co. Beijing, China), performed following the procedures recommended by the manufacturer. Specimens found to have an absorbance level lower than the cut-off value were considered negative. To confirm the positive specimen, specimens with an absorbance level greater than the cut-off value were rerun in duplicate. Samples consistently positive were identified as HBsAg seropositive, while those re-assayed but fell below the cut-off value were considered negative [27].

#### HCV testing

Anti-HCV immunoglobulin G (IgG) antibody was tested using an ELISA kit to determine HCV infection status according to the manufacturer’s protocol (Wantai Biomedical, Beijing, China). All the plasma samples were blindly assayed in duplicate [28].

#### Syphilis testing

Plasma samples were tested using a rapid plasma reagent test (Span Diagnostics Ltd, Surat, India), and results were confirmed by the *Treponema pallidum* hemaglutination assay (TPHA, Syphagen TPHA, Biokit, Spain) [26].

All the above serological tests were performed by the same two experienced technicians according to the manufacturers' standard protocols. Duplicate negative, positive, and blank controls were always analyzed in parallel.

### Statistical analysis

Original questionnaires and laboratory testing results were entered into EpiData3.1 and audited for accuracy, then transferred to an SPSS database for further management. Frequency and percentage were used for categorical variables, with mean and standard deviation (SD) for continuous variables. Tests of associations between ways of seeking sex partners and sexual behaviors were based on the chi-square test or Fisher’s exact test, whichever was appropriate. Differences for ordinal categorical variables were tested using Cochran Armitage tests. Potential correlates of KSHV seropositivity were assessed by using logistic regression models. Adjusted odds ratios (aORs) and corresponding 95% CIs were calculated and used to assess the determinants of KSHV infection. *P*-values less than or equal to 0.05 were considered statistically significant. The R programming language (version 4.1.1) was used for all statistical analyses.

## Results

### Sociodemographic characteristics and associations with KSHV seropositivity

Finally, a total of 520 MSMs were enrolled in the study, of which 228 (43.8%) were KSHV seropositive. The mean age of the participants was 30.7 (SD = 8.3) (Table 1). 80.8% of them had received a college education or above. 74.4% were single, divorced or separated. Individuals with a higher level of monthly income had lower KSHV seroprevalence (Chi-sqaure_*trend*_ = 4.11, *P* = 0.043), and such a significant association was also observed in our multivariable analysis. Furthermore, compared with non-student adults, KSHV seroprevalence was higher among students (aOR = 1.96; 95%CI: 1.09–3.61).

**Table 1:** Sociodemographic characteristics and their associations with KSHV seropositivity among MSM

Variables	No. (%)	Seropositive (%)	ORs (95%CI)	aORs (95%CI)^a^
Age group (years)				
18–25	153 (29.4)	70 (45.8)	1.00	1.00
26–35	252 (48.5)	117 (46.4)	1.03 (0.69–1.54)	1.02 (0.68–1.53)
≥ 36	115 (22.1)	41 (35.7)	0.66 (0.40–1.08)	0.73 (0.43–1.22)
Ethnicity				
Han	487 (93.7)	216 (44.4)	1.00	1.00
Others	33 (6.3)	12 (36.4)	0.72 (0.34–1.49)	0.71 (0.33–1.46)
Education level				
Senior high and below	100 (19.2)	34 (34.0)	1.00	1.00
College and above	420 (80.8)	194 (46.2)	1.67 (1.06–2.63)*	1.51 (0.95–2.45)
Residency				
Local	174 (33.5)	77 (44.3)	1.00	1.00
Non-local	346 (66.5)	151 (43.6)	0.98 (0.68–1.41)	0.97 (0.66–1.44)
Marital status				
Single	295 (56.7)	136 (46.1)	1.00	1.00
Married/cohabiting	133 (25.6)	47 (35.3)	0.64 (0.42–0.98)*	0.73 (0.46–1.14)
Divorced/separated	92 (17.7)	45 (48.9)	1.12 (0.70–1.79)	1.18 (0.73–1.90)
Occupation				
Non-student	461 (88.7)	193 (41.9)	1.00	1.00
Student	59 (11.3)	35 (59.3)	2.03 (1.17–3.51)*	1.96 (1.09–3.61)*
Monthly income (*P* = 0.043^b^)				
≤ 2999	54 (10.4)	31 (57.4)	1.00	1.00
3000–5999	115 (22.1)	52 (45.2)	0.61 (0.32–1.18)	0.68 (0.34–1.34)
6000–9999	136 (26.2)	58 (42.6)	0.55 (0.29–1.04)	0.50 (0.26–0.97)*
≥ 10,000	215 (41.3)	87 (40.5)	0.50 (0.28–0.92)*	0.40 (0.20–0.78)*
Ways of seeking sex partners				
Online	455 (90.1)	197 (43.3)	1.00	1.00
Offline	50 (9.9)	22 (44.0)	1.03 (0.57–1.85)	1.13 (0.62–2.06)

*OR* odds ratio, *aORs* adjusted odds ratios, *CI* confidence interval

^a^Adjusted for age, ethnicity, and education level

^b^Using Cochran Armitage tests to test the linear association between monthly income and KSHV seroprevalence

^*^The results were statistically significant (*P*-value < 0.05)

Interestingly, the results showed that 90.1% of participants sought male sex partners via the Internet rather than offline (Table 1). We further analyzed the potential impacts due to different ways of seeking sex partners and found a trend toward increased incidence of risky sexual behaviors among the participants who sought sex partners online. Compared with individuals seeking sex partners offline, those who sought via the Internet were more likely to report multiple (≥ 2) male sex partners (*P* = 0.019) and recreational drug use (*P* = 0.008) in the past six months (data not shown).

### Co-infections and associations with KSHV seropositivity

MSMs are at high risk of a variety of pathogens. In the present study, the seroprevalence of HIV, HBV, HCV, and syphilis were 13.3%, 10.8%, 4.2%, and 5.4%, respectively. Overall, infections with more than one pathogen were 59.0%. Dual infection with KSHV was 12.1% (data not shown). Multivariate analyses revealed that participants infected with HCV (aOR = 2.61; 95%CI: 1.05–7.10) or syphilis (aOR = 2.91; 95%CI: 1.30–6.89) were more likely to be infected with KSHV, while co-infection with HIV or HBV was not significantly associated with an increased risk of KSHV seropositivity (Table 2).

**Table 2:** Serostatus of HIV, HBV, HCV, and syphilis and their associations with KSHV seropositivity among MSM

Variables	Seropositive (%)	ORs (95%CI)	aORs (95%CI)^a^
HIV			
Negative	197/451 (43.7)	1.00	1.00
Positive	31/69 (44.9)	1.05 (0.63–1.75)	1.17 (0.69–1.96)
HBV			
Negative	207/464 (44.6)	1.00	1.00
Positive	21/56 (37.5)	0.74 (0.42–1.32)	0.76 (0.42–1.35)
HCV			
Negative	213/498 (42.8)	1.00	1.00
Positive	15/22 (68.2)	2.87 (1.15–7.16)*	2.61 (1.05–7.10)*
Syphilis			
Negative	210/492 (42.7)	1.00	1.00
Positive	18/28 (64.3)	2.42 (1.09–5.34)*	2.91 (1.30–6.89)*

*OR* odds ratio, *aORs* adjusted odds ratios, *CI* confidence interval

^a^Adjusted for age, ethnicity, education level and monthly income

^*^The results were statistically significant (*P*-value < 0.05)

### Sexual behaviors and associations with KSHV seropositivity

**Fig. 1:**
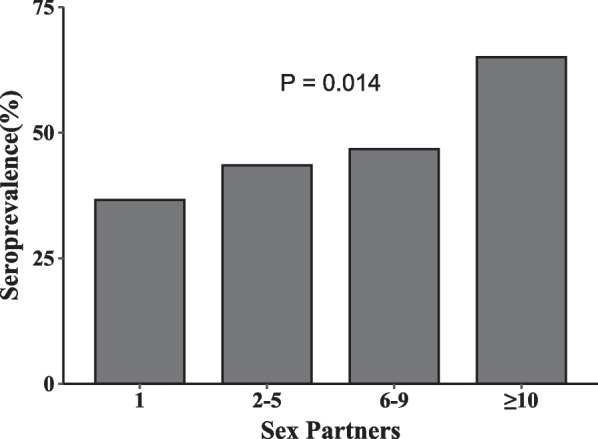
The distribution of KSHV seroprevalence among participants with different numbers of male sex partners

Sexual behaviors also play important roles in KSHV acquisition. In this study, 90.8% (472/520) of all subjects had sex with men in the past six months, of which 67.6% reported having had two or more male sex partners, and 40.7% had used condoms inconsistently. 42.1% of all subjects have ever used recreational drugs before or during sex. The multivariable logistic regression adjusting for age, ethnicity, education level, and monthly income indicated that participants with multiple male sex partners were more likely to be infected with KSHV (aOR = 1.56; 95%CI: 1.03–2.38) compared with those monogamous individuals (Table 3). After further analysis, we found a significant positive linear association between more male sex partners and higher KSHV seroprevalence (Chi-sqaure_*trend*_ = 6.06, *P* = 0.014) (Fig. 1).

**Table 3:** Sexual behaviors in the past 6 months and their associations with KSHV seropositivity among MSM

Variables	Seropositive (%)	ORs (95%CI)	aORs (95%CI)^a^
Forms of sex			
Oral sex	21/47 (44.7)	1.00	1.00
Anal sex	16/32 (50.0)	1.24 (0.50–3.05)	1.77 (0.69–4.59)
Both	163/393 (41.5)	0.88 (0.48–1.61)	1.00 (0.53–1.90)
Role during anal sex			
Insertive	61/154 (39.6)	1.00	1.00
Receptive	54/116 (46.6)	1.33 (0.82–2.16)	1.19 (0.71–1.98)
Both	85/202 (42.1)	1.11 (0.72–1.70)	1.01 (0.65–1.59)
Male sex partners			
1	56/153 (36.6)	1.00	1.00
≥ 2	144/319 (45.1)	1.43 (0.96–2.12)	1.56 (1.03–2.38)*
Condom use for anal sex with man			
Inconsistently	76/192 (39.6)	1.00	1.00
Consistently	124/280 (44.3)	1.21 (0.84–1.76)	1.17 (0.80–1.72)
Ever had sex with woman			
No	205/450 (45.6)	1.00	1.00
Yes	23/70 (32.9)	0.58 (0.34–1.00)*	0.62 (0.36–1.07)
Used saliva as a lubricant during anal sex			
No	129/272 (47.4)	1.00	1.00
Yes	99/248 (39.9)	0.74 (0.52–1.04)	0.77 (0.53–1.10)
Ever used recreational drug			
No	131/301 (43.5)	1.00	1.00
Yes	97/219 (44.3)	1.03 (0.73–1.47)	1.09 (0.75–1.56)

*OR* odds ratio, *aORs* adjusted odds ratios, *CI* confidence interval

^a^Adjusted for age, ethnicity, education level and monthly income

^*^The results were statistically significant (*P*-value < 0.05)

## Discussion

Understanding the prevalence and correlates of KSHV infection among MSM is particularly important for designing proper interventions for curbing KSHV transmission. In the present study, an alarmingly high seropositivity of KSHV (43.8%) among MSM was observed. Given the fact that the BC-3 cells have a high level of lytic antigens after being stimulated, this could increase the sensitivity but may also result in false-positives in our study [29]. Therefore, we adjusted the result according to the sensitivity and specificity of the lytic IFA test that have been reported [29]. The adjusted prevalence of KSHV was 29.8% (Additional file 1), higher than other high-risk groups in China [27, 28] and similar to the MSM population in previous studies [17, 26], indicating that the KSHV infection in MSM remains a significant public health concern.

Co-infection with multiple infectious agents is common among MSM. The positive association between syphilis and KSHV infection in our study is consistent with previous studies, implying that the two agents may share similar transmission modalities or facilitate the acquisition of each other in this population [14, 17]. A trend toward increased risk of KSHV infection with HCV co-infection was also observed in this study. However, there are conflicting findings [17]. Differences in sample size and characteristics of participants were likely to explain the inconsistent observations. Based on previous evidence of HCV sexually transmitted among MSM, high-risk sexual practice might exert a critical part in KSHV/HCV coinfection in this population [30, 31]. It is well documented that KSHV infection was strongly associated with HIV infection [14]. Surprisingly, such a significant association was not observed in this study, and the seroprevalence of KSHV is comparable in HIV-positive and HIV-negative participants (44.9% *vs.* 43.7%). Studies have reported no association between KSHV seropositivity and peripheral blood CD4 cell count in HIV-infected individuals [32, 33]. Therefore, the role of HIV infection or associated immunosuppression in KSHV infection might be limited. Notwithstanding, further studies will be necessary to explain this phenomenon.

There is good evidence that KSHV infection is associated with risky sexual behaviors among MSM [15, 34]. The linear positive association between KSHV infection and the number of recent male sex partners in this study was indicative of the transmission route of this virus through intimate contact among men [20]. We did not detect a significant association between KSHV infection and the use of saliva as a lubricant in anal sex. However, subjects might also have had kisses or oral sex, which provided opportunities for saliva contact. Therefore, disentangling saliva contact from sexual contact during sexual intimacy practice was a pretty challenging task in our study, and the possibility of saliva transmission for KSHV cannot be ruled out. Consistent with previous studies, the results showed that heterosexual contact seemed to be ineffective for KSHV acquisition among MSM [17]. However, it cannot be ignored that MSM are a potential source of KSHV transmission to the heterosexual population. Of note, our study also supported the idea that the increasing popularity of dating apps could potentially increase high-risk sexual behaviors [35, 36]. Its subsequent impact on the transmission of infectious agents, including KSHV, warrants further studies.

Another key finding from our study is that compared with non-student adults, the risk among student MSM was increased about twofold (aOR = 1.96). Student MSM are sexually active and have become a high-risk population for HIV infection [37]. However, few studies have focused on the risk of KSHV infection among this vulnerable population, which clearly deserves more attention. The results also suggest that a higher level of monthly income is a protective factor for KSHV infection. It is possible that individuals with higher economic levels tend to focus more on self-protection [38].

Our study had several limitations. First, the study subjects were at risk of selection bias due to non-random venue-based and snowball sampling strategies for recruitment and may not represent the general MSM population. Second, social desirability and recall bias may have compromised the accuracy of the answers to the sexual behaviors. Third, the seroprevalence of KSHV was likely overestimated in our study. We adjusted the prevalence, and the result showed a relatively high burden of KSHV infection. Finally, the cross-sectional design limited interpretability and more related longitudinal research is necessary.

## Conclusion

Collectively, the prevalence of KSHV remains high in MSM in Shanghai, China. Our findings provide further evidence that having multiple male sex partners is a risk factor for KSHV infection among this population. In addition, the high prevalence of KSHV in student MSM requires more attention. Given the potentially serious consequences, targeted intervention for KSHV infection and co-infections is needed among MSM.

## Supplementary Information

**Additional file 1: Table S1**. Contingency table of counts based on the diagnostic test and the true state of KSHV infection.

## Data Availability

The datasets generated and analyzed during the current study are not publicly available due to their proprietary nature, privacy, and ethical concerns, but are available from the corresponding author on reasonable request.

## Abbreviations

KSHV: Kaposi’s sarcoma associated herpesvirus
MSM: Men who have sex with men
HHV-8: Human herpesvirus type 8
KS: Kaposi’s sarcoma
PEL: Primary effusion lymphoma
MCD: Multicentric Castleman’s disease
KICS: KSHV-associated inflammatory cytokine syndrome
STIs: Sexually transmitted infections
HIV: Human immunodeficiency virus
HBV: Hepatitis B virus
HCV: Hepatitis C virus
IFA: Immunofluorescence assay
TPA: Tetradecanoyl phorbol acetate
ELISA: Enzyme-linked immunosorbent assay
IgG: Immunoglobulin G
TPHA: *Treponema pallidum* Hemaglutination assay
OR: Odds ratio
aOR: Adjusted odds ratio
CI: Confidence interval
RMB: Renminbi
