# Supplementary material for: Seroprevalence and risk factors for Kaposi’s Sarcoma associated herpesvirus among men who have sex with men in Shanghai, China
Source: BMC Infect Dis. 2023 Jan 31;23:59. doi: 10.1186/s12879-023-08028-y (PMC9887846; doi:10.1186/s12879-023-08028-y)
Supplement: Supplementary file 1 — Additional file 1: Table S1. Contingency table of counts based on the diagnostic test and the true state of KSHV infection. [file 12879_2023_8028_MOESM1_ESM.docx]

**Additional file 1**

**Seroprevalence and Risk Factors for Kaposi’s Sarcoma Associated Herpesvirus among Men Who Have Sex with Men in Shanghai, China**

Yi Li^1^, Xingcan Zhang^2^, Yue Zhang^3^, Minqi Wei^3^, Sijie Tao^3^, Ying Yang^3,^*

1. Department of Epidemiology, School of Public Health, Fudan University

2. Department of Gynecology, Obstetrics and Gynecology Hospital, Fudan University, Shanghai, 200011, China；

3. Department of HIV/AIDS prevention, Center for Disease Control and Prevention of Minhang District, Shanghai, 201101, China；

**Supplementary material: the adjustment of KSHV infection prevalence**

We constituted a contingency table of counts based on the diagnostic test and the true state of KSHV infection. As shown in Table S1, the “P” represents the true prevalence. The sensitivity and specificity were reported previously [1]. The detailed calculation process is presented in Table S1 and below. After calculation, the adjusted prevalence was 29.8%.

Table S1. Contingency table of counts based on the diagnostic test and the true state of KSHV infection

| Test | True state of infection | | Total |
| --- | --- | --- | --- |
|  | + | - |  |
| + | 520 × P × sensitivity | 520 × (1-P) × (1- specificity) | 228 |
| - | 520 × P × (1- sensitivity) | 520 × (1-P) × specificity | 292 |
| Total |  |  | 520 |

P: the true prevalence of KSHV; sensitivity=100%; specificity=80%

Substitute the known values into the equation:

520 × P × 100% + 520 × (1-P) × (1-80%) = 228

This calculation has yielded a P of 29.8%.

Reference

1. Cohen A, Wolf D, Guttman-Yassky E, Sarid R: **Kaposi's sarcoma-associated herpesvirus: Clinical, diagnostic, and epidemiological aspects**. *Crit Rev Clin Lab Sci.* 2005; **42**:101-53.
